# Hypertension in Metabolic Syndrome: Vascular Pathophysiology

**DOI:** 10.1155/2013/230868

**Published:** 2013-03-20

**Authors:** Yolanda Mendizábal, Silvia Llorens, Eduardo Nava

**Affiliations:** Department of Medical Sciences, University of Castilla-La Mancha, School of Medicine and Regional Centre for Biomedical Research (CRIB), 02006 Albacete, Spain

## Abstract

Metabolic syndrome is a cluster of metabolic and cardiovascular symptoms: insulin resistance (IR), obesity, dyslipemia. Hypertension and vascular disorders are central to this syndrome. After a brief historical review, we discuss the role of sympathetic tone. Subsequently, we examine the link between endothelial dysfunction and IR. NO is involved in the insulin-elicited capillary vasodilatation. The insulin-signaling pathways causing NO release are different to the classical. There is a vasodilatory pathway with activation of NO synthase through Akt, and a vasoconstrictor pathway that involves the release of endothelin-1 via MAPK. IR is associated with an imbalance between both pathways in favour of the vasoconstrictor one. We also consider the link between hypertension and IR: the insulin hypothesis of hypertension. Next we discuss the importance of perivascular adipose tissue and the role of adipokines that possess vasoactive properties. Finally, animal models used in the study of vascular function of metabolic syndrome are reviewed. In particular, the Zucker fatty rat and the spontaneously hypertensive obese rat (SHROB). This one suffers macro- and microvascular malfunction due to a failure in the NO system and an abnormally high release of vasoconstrictor prostaglandins, all this alleviated with glitazones used for metabolic syndrome therapy.

## 1. Introduction 

**Figure 1:**
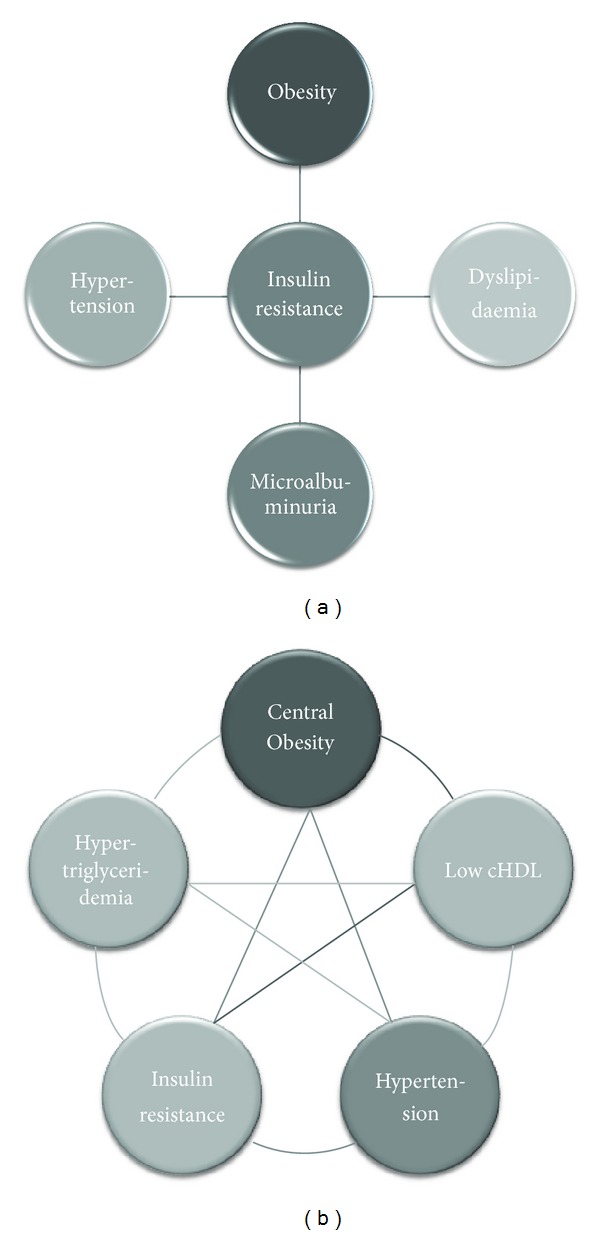
Two ways to conceptualize metabolic syndrome and the position hypertension and the other symptoms occupy. According to the WHO definition, insulin resistance is central to any other symptom (a). Others define metabolic syndrome as a cluster of symptoms where none has a central position (b).

The metabolic syndrome is a cluster of metabolic and cardiovascular symptoms that are strongly associated with type II diabetes mellitus. In this kind of diabetes, rather than prolonged high levels of glycemia, there is insulin resistance with secondary hyperinsulinemia, both very frequently associated with, hypertension, dyslipemia, atherosclerosis, and, most importantly, obesity (Figure 1) [1]. Vascular disorders are central to this condition. Quoting prof. Yki-Järvinen “…after all, from a clinical point of view, type II diabetes mellitus is a disease of blood vessels, not muscle.” [2]. For these reasons, it is also known as cardiometabolic syndrome [1], and hypertension plays a pivotal role. Indeed, risk estimates according to the Framingham study show that roughly 80% of essential hypertension in men and 65% in women can be directly attributed to obesity [3]. There is a clear association between body mass index and arterial pressure even in nonobese, lean people [4–6]. Still, some obese people are not hypertensive. For example, the North American Pima Indians, who have a high prevalence of obesity, but do not have corresponding high rates of hypertension [7].

The history of metabolic syndrome takes us back to the early 20th century, when two physicians, the Swedish, Kylin and the Spanish Marañón nearly simultaneously and independently published in the journal *Zentralblatt für Innere Medizin* two articles under almost the same title: *Über Hypertonie und Zuckerkrankheit *[8, 9]. In these articles, the two physicians described for the first time the coexistence of hypertension and diabetes mellitus in adults and proposed a common mechanism for the development of these disorders. In 1988, Reaven, hypothesized that insulin resistance is the common etiological factor of a group of disorders, such as high blood pressure, hyperinsulinemia, high levels of low density lipoproteins (LDL), triglycerides, and cholesterol, and low levels of high density lipoproteins (HDL). Reaven named this collection of disorders “syndrome X” [1]. A year later, Kaplan added to the pathologies described by Reaven a very important factor, central adiposity (increase in splanchnic and subcutaneous fat depots in the abdominal region) [10]. Since then, abdominal obesity has been considered one of the typical components of the syndrome.

Both type 2 diabetes mellitus and metabolic syndrome are reaching epidemic proportions. Considering that 220 million people worldwide are diabetic, this disease has become a serious epidemiological problem [11]. The problem is not only the size of the figures but also the alarming increase in only a few decades (46% in the 1990s). Metabolic syndrome is, probably, the most important challenge for health authorities in developed and developing countries [11, 12]. In Europe there is a clear North-South gradient in almost all cardiovascular risk factors related with metabolic syndrome. For example, mortality from coronary heart disease, expressed as a mortality ratio, presented in men aged 30–69 the following geographical indices: 8.2 Iceland; 5.1 England; 2.2 Italy; 1.8 Spain; and 0.9 Portugal [13]. However, there is no doubt that the paradigm of overdevelopment-overweight is the United States. With the turn of the century, 61% of Americans were sufficiently overweight to suffer health problems directly derived from this condition [14]. A diet that is as excessive as inadequate has yielded these epidemiological figures in less than 20 years: between 1977 and 1995 daily caloric intake rose by 200 calories. This is the equivalent to an increment of 10 calories per year [14].

## 2. Role of the Sympathetic Nervous System

There are 3 conditions, typical of metabolic syndrome, that may cause an exacerbation of sympathetic tone. Namely, hyperinsulinemia, hyperleptinemia, and hyperlipidemia. In 1981, it was reported that hyperinsulinemia, independently of changes in glycemia, caused a substantial increase in circulating noradrenaline concentration accompanied by an increase in blood pressure [15]. These sympathoexcitatory effects of insulin appear to be centrally mediated, since they are apparent only during systemic insulin infusion but not local infusion [16]. In addition, high levels of insulin increase sodium reabsorption [17] favouring expansion of extracellular fluid volume, which may predispose to hypertension [18]. Furthermore, obesity impairs renal-pressure natriuresis and causes sodium retention. Obese subjects require increased arterial pressure to maintain sodium balance, indicating impaired renal-pressure natriuresis [19]. 

In addition to insulin, leptin can also be a link between obesity and increased sympathetic activity. Besides its effect on appetite and metabolism, leptin acts in the hypothalamus to increase blood pressure through activation of the sympathetic nervous system [20]. High circulating levels of leptin are reported to explain much of the increase in the renal sympathetic tone observed in obese human subjects [21]. Leptin-induced increases in renal sympathetic activity and blood pressure are mediated by the ventromedial and dorsomedial hypothalamus [22].

Finally, high circulating levels of free fatty acids in visceral obese individuals may participate in the activation of the sympathetic nervous system. The increased release of free fatty acids into the portal vein from lipolysis in visceral fat depots could explain the strong association between visceral obesity and increased sympathetic nerve outflow [23].

## 3. Role of Insulin

### 3.1. Insulin Resistance and Endothelial Dysfunction

In 1939, Himsworth postulated that type 2 diabetes mellitus was not only an insulin deficiency state but also a disease in which cells are unresponsive to insulin. Thus, Himsworth's work gave birth to the concept of insulin resistance [24, 25]. Insulin resistance is clinically defined as the inability of a known quantity of insulin (exogenous or endogenous) to increase glucose uptake and utilization in an individual as much as it does in a normal population [26]. There is a clear link between endothelial dysfunction and insulin resistance [27, 28] but the mechanism by which insulin resistance leads to endothelial dysfunction is complex and involves the action of mediators of inflammation in the visceral fat, liver, and muscle [29]. It is well known that insulin resistance and compensatory hyperinsulinaemia, besides activating the mechanisms mentioned above, have also a vascular toxicity effect, mainly at the endothelial level. This, partly because insulin resistance impairs the production of NO, favors the production of endothelin-1 and the vasoconstrictive and mitogenic responses on the vascular wall [30].

#### 3.1.1. Role of NO in Insulin Resistance

King and Johnson reported in 1985 that the endothelial cell membrane displays insulin receptors [31]. Functional studies indicate that endothelium-derived NO is involved in the insulin-elicited increase in blood flow and recruitment of capillaries that physiologically links hemodynamics to the metabolic action of insulin on the tissues [32–34]. Insulin resistance is associated with impaired NO synthase activity [35] and an abnormal basal NO-mediated dilation in the forearm arterial bed [36]. The insulin-induced increase of microvascular endothelium-dependent vasodilation is abolished in insulin resistance conditions such as obesity [37]. Moreover, insulin has been shown to constrict rather than dilate forearm resistance arteries in obese patients [38]. On the other hand, inhibition of NO synthesis or endothelium removal reveals a vasoconstrictor effect of insulin on isolated arterioles [39]. Definitive proof of the relationship between NO and insulin sensitivity has been provided by knock-out mice that are homozygous null for the eNOS gene. These peculiar animals display an expected hemodynamic phenotype of increased basal blood pressure but also are insulin resistant [40]. Therefore, insulin has indeed a hemodynamic component, albeit small compared to the metabolic one. But both are coupled in such a manner that endothelial dysfunction can cause insulin resistance, and this, in a vicious circle, aggravates endothelial function. 

Interestingly, insulin-signaling pathways in vascular endothelium leading to the activation of endothelial NO synthase are completely independent and distinct from classical calcium-dependent mechanisms used by G-protein-coupled receptors, such as the acetylcholine receptor [34]. The messenger pathway that is activated when insulin binds insulin receptor appears to be as follows [41]: insulin binds insulin receptor (INS-R) which is at the same time a tyrosine kinase and this undergoes autophosphorylation of tyrosine residues. INS-R phosphorylates insulin receptor substrate-1 (IRS-1). The signalling pathway from insulin branches at IRS-1. One of the branches involves the activation of phosphoinositide 3 kinase (PI-3K), leading to phosphatidylinositol-3,4,5-triphosphate as well as to phosphorylation and activation of phosphoinositide-dependent kinase 1 (PDK-1). Both products, in turn, phosphorylate and activate Akt (also called protein kinase B, PKB). Akt directly phosphorylates eNOS at Ser^1177^, resulting in increased eNOS activity and NO production [42]. Remarkably, the vascular actions of insulin that stimulate the production of NO possess remarkable similarities to metabolic insulin-signaling pathways. For instance, activation of Akt is also a common step for glycogen synthase kinase inhibition and GLUT-4 transporter translocation [41].

#### 3.1.2. Role of Endothelin-1 in Insulin Resistance

In 1991, Oliver et al. demonstrated that insulin was able to stimulate endothelin-1 (ET-1, a very strong vasoconstrictor) gene expression in endothelial cells [43]. Later, it was shown that insulin can modulate circulating ET-1 levels [44] and increased plasma levels of ET-1 were observed in type II diabetic patients [45]. An additional work in the skeletal muscle circulation reported that insulin stimulates both NO activity (already known as we showed before) and ET-1 [46].

The authors then suggested that an imbalance between the release of both substances may be involved in pathophysiology of hypertension and atherosclerosis in insulin-resistant states associated with endothelial dysfunction [46]. Following research has shown that insulin induces endothelin-mediated vasoconstriction only when NO synthase or phosphatidylinositol-3 kinase (PI3K) is inhibited [47]. In a paper elegantly entitled “Endothelin antagonism uncovers insulin-mediated vasorelaxation in vitro and in vivo” [48], Verma et al. demonstrated that insulin-mediated vasorelaxation is only well patent when antagonizing ET-1 receptors. This proved previous proposals that insulin exhibits a dual and opposite action on blood vessels: NO-mediated vasodilation and ET-1-mediated vasoconstriction. It is known that MAPK activation by IRS-1 causes the release of endothelin-1, which promotes insulin resistance (by reducing blood supply to the skeletal muscle), increases oxidative stress, reduces the bioavailability of NO, and promotes a proatherogenic state [49]. 

### 3.2. Hyperglycaemia and Vascular Function

Regardless of the evidence linking the vascular dysfunction of type II diabetes mellitus with failures in the vascular biology of insulin, there are many reports that attribute these dysfunctions to the very fact of the existing hyperglycaemia. We wish to draw attention to the functional effects of the acute excess in glucose occurring in a particular moment. In this regard, it has been reported that glucose favours vasoconstriction [50] and impairs vasodilation [51]. In arteries of diabetic rats, Taylor et al. demonstrated that hyperglycaemia reduces the tonic release of NO [52] and established a central role for glucose in the development of vascular functional changes associated with experimental diabetes [50]. Most interesting is the finding that in healthy subjects, acute hyperglycaemia impairs endothelium-dependent vasodilation in both the microcirculation and the macrocirculation when assessed in the brachial artery [53]. More precise data on the mechanisms involved in hyperglycaemia was released by Sobrevia et al. [54] who showed that exposure of endothelial cells to elevated glucose was associated with stimulation of L-arginine transport paralleled by an increase in basal release of NO and prostacyclin. This would be good news if they did not find as well that insulin treatment downregulated the elevated activity of the L-arginine transport system and that of NO synthase in the cells exposed to hyperglycaemia. They concluded that the modulation of the human endothelial cell L-arginine-NO pathway by insulin is influenced by predisposing hyperglycaemic clinical conditions [54]. In a later study, Renaudin et al. demonstrated that the vasodilatory effect of insulin disappears when hyperglycaemia exists, perhaps blunted by the vasoconstrictive effect of glucose [55].

### 3.3. Insulin Actions on Blood Pressure: The Insulin Hypothesis of Hypertension

So far we have focused on the cardiovascular effects of insulin at a local level. However, it cannot be forgotten that insulin has systemic actions affecting the sympathetic nervous system and kidney. The surge of epidemiological reports relating insulin resistance and hyperinsulinemia has fueled the idea of the so-called insulin hypothesis of hypertension. There is no question that insulin resistance is epidemiologically linked with hypertension [1]. The insulin hypothesis of hypertension proposes that the compensatory hyperinsulinemia that occurs with insulin resistance increases sodium reabsorption and sympathetic activity, which combine to cause elevated arterial pressure. Support for this hypothesis comes from various lines of evidence. First, the correlation between insulin resistance and high blood pressure [56], which is emphasized by the fact that, even lean individuals with essential hypertension, display insulin resistance and hyperinsulinemia. Some go a step further asserting that essential hypertension is “per se” an insulin resistance state [57]. Second, as explained before, insulin has multiple actions on the sympathetic nervous system, the kidney, and the vasculature which can lead to hypertension. Third, the observation that drugs which improve insulin resistance and decrease hyperinsulinemia, are reported to be antihypertensive. For instance, Landin et al. reported that oral administration of metformin to insulin-resistant, hypertensive men increased insulin sensitivity and significantly decreased arterial pressure [58]. Another remarkable example is the well-known blood pressure lowering effects of insulin sensitizers glitazones [59]. For review, see [60]. Fourth and finally, the observation that some antihypertensives, such as angiotensin II converting enzyme inhibitors [61] or angiotensin II receptor antagonists [62], increase insulin sensitivity as well. Despite the size of the support in favour of the insulin hypothesis of hypertension, there is also important evidence against. For instance, the eminent physiologist Hall and his collaborators failed to find a correlation between insulin and hypertension in a well-controlled model in dogs [63].

## 4. Role of Adipokines

Traditionally, adipocytes were considered energy reservoirs that store triglycerides during feeding and deliver fatty acids during fasting. However, it has become quite clear that adipose tissue does much more than this and is responsible for the synthesis and secretion of numerous proteins. The first protein described was adipsin [64]. Later, the secretion of cytokines such as TNF-*α* was described [65], thus conferring immune functions to adipocytes. Funahashi et al. named these substances adipocytokines [66]. Undoubtedly, the most relevant discovery was leptin by the Friedman group in 1994 [67]. Because the vast majority of substances produced by the adipocyte are not necessarily cytokines, Trayhurn and Wood recommended the term *adipokines* instead. Therefore, adipokines are defined as any substance synthesized and secreted by the adipocytes [68]. Thus, it has become quite clear that adipose tissue is indeed an endocrine organ. In fact, it can be the largest organ in the body. This is physiologically and pathophysiologically important because the total amount of secreted adipokines are enormous and may affect the whole body economy, especially considering that every adipocyte is connected to the vascular network [69]. It is well known that dysregulation of the production and secretion of adipokines is involved in the development of metabolic and cardiovascular diseases. In metabolic syndrome, intra-abdominal visceral fat accumulation has been shown to play a key role in the development of a variety of metabolic and circulatory disorders through the dysregulation of adipokine secretion [70].

### 4.1. Perivascular Adipose Tissue and Vascular Function

The function of adipose tissue as an endocrine organ has important implications in the understanding of the pathophysiological relationships between excess body fat and hypertension. Almost all the systemic arteries are surrounded by a layer of perivascular adipose tissue (PVAT). In the majority of myographic studies, PVAT is removed on a routine basis. This is a custom based on the assumption that PVAT can prevent the diffusion of vasoactive substances. This is perhaps the reason that, despite the ubiquity of PVAT, very little is known about its function in vascular biology. Perivascular fat certainly has a modulator action on vascular contractility. This was described by Soltis and Cassis in a study published in 1991 [71]. This work has often been misinterpreted as the first postulator of a supposed prorelaxing role of PVAT. These researchers describe a decrease in the sensitivity to noradrenaline when aortic segments remain with PVAT. They demonstrate that this is due to the uptake and elimination of this catecholamine by adipose tissue. They postulate that the nerve endings within PVAT recapt and remove noradrenaline within the synaptic gap. This obviously results in a buffered effect of this neurotransmitter, but it is not postulated that PVAT releases any anticontractile factor.

In more recent years, several groups have dealt with the possible vasoactive role of PVAT. The group of González et al. has been especially interested in the vasoactive properties of the tunica adventitia, to which they attribute a role in the contractile ability of the responses modulated by the endothelium [72]. Later on, Gao et al. as well as Rey et al. claimed that PVAT promotes the vasoconstrictor response to electrical stimulation [73] and impaired endothelial function [74] via reactive oxygen species generated by NADPH oxidase. On the other hand stands the work pursued by Gollasch's group and initiated by Löhn et al. who claim to have found a diffusible factor derived from PVAT, which they called “adventitium-derived relaxing factor” or ADRF [75]. In a following paper, the “A” standed for “adipocyte” instead [76]. A relevant amount of literature has confirmed the existence of this anticontractile diffusible substance (see [77] for review). Still, there is no unanimity regarding the nature and mechanism of action of ADRF. For Verlohren and coworkers, it is independent from the endothelium [76], but not for Gao et al. [78]. What seems clear is that the vasodilatory effect of ADRF is mediated by the opening of different K^+^ channels on vascular smooth muscle cells [75, 76, 78–80]. Endocrine and vascular paracrine functions of a variety of adipokines are shown in Table 1. We shall focus on those with particular vasoactive actions, namely, leptin, adiponectin, TNF-*α*, prostaglandins, angiotensin II, and endothelin-1.

### 4.2. Leptin

The discovery that the endothelium expresses the leptin receptor OB-Rb [81], converted endothelial cells, just like those of the hypothalamus, in a target for this hormone. The presence of leptin receptors in the vascular endothelium and not only in the central nervous system is important because it allows to find a link between leptin and altered vascular function in obesity [82]. Leptin is an NO-dependent vasodilator but also increases peripheral vascular resistance and sympathetic nerve activity [83]. The concentration of plasma leptin is correlated with adiposity, and hyperleptinemia is indeed considered an independent cardiovascular disease risk factor [84]. There are two theories that relate leptin's cardiovascular effects to obesity. One of them proposes that leptin is involved in the control of vascular tone simultaneously causing a neurogenic pressor action and an opposite depressor effect mediated by NO [85]. Another theory, based on experiments performed in coronary arterioles [86], proposes that, paradoxically, leptin causes itself NO-dependent vasodilation and, at the same time, its very presence impairs endothelium-dependent relaxations, that is, produces endothelial dysfunction. The problem with this interesting theory is that leptin-induced relaxation occurs at concentrations well above those found in very obese subjects. Physiological (lean) or pathophysiological concentrations (obese) of leptin have, however, little direct effect on vascular tone. Possibly, the most relevant aspect of this theory is that leptin concentrations actually existing in obese patients do elicit endothelial dysfunction [86].

### 4.3. Adiponectin

Adiponectin is the secretory protein produced in largest amounts by adipocytes and present in high and stable concentration in the plasma. In healthy subjects, adiponectin carries out its roles preventing the development of vascular changes and has been reported to be associated with lipid metabolism [87], glucose metabolism [88], and insulin resistance [89]. Unlike leptin, plasma adiponectin levels are negatively correlated with body mass index. This negative correlation is stronger between adiponectin levels and visceral adiposity than between the protein and subcutaneous adiposity [90]. Also, there is a close relationship between low concentrations of adiponectin in the blood, insulin resistance, and hyperinsulinemia. It has been suggested that the decrease in plasma adiponectin concentration contributes to the metabolic complications associated with obesity [91]. Adiponectin improves NO-dependent vasodilation by opening voltage-dependent potassium channels [92–94].

Some reports suggest that adiponectin plays an important role in insulin actions and hypoadiponectinemia may result in insulin resistance and diabetes mellitus. In fact, Lindsay et al. demonstrated that plasma levels of adiponectin were lower in Pima Indians, a unique cohort with high prevalence of obesity [95]. They also demonstrated that plasma levels of adiponectin are strongly correlated with insulin sensitivity evaluated by glucose disposal rate [96]. The study of the Pima Indian population demonstrates that adiponectin may play a crucial role in the development of diabetes mellitus and that high adiponectin levels should protect from the deterioration of glucose metabolism. Thus, hypoadiponectinemia could be a significant background of vascular changes and metabolic disorders, including insulin resistance and, possibly, a background for hypertension as well. Indeed, some studies show that hypertensive subjects have lower levels of plasma adiponectin [97]. 

### 4.4. Tumor Necrosis Factor-*α* (TNF-*α*)

Since Hotamisligil's group reported that adipose tissue expresses TNF-*α*, one of the candidate molecules inducing insulin resistance adipokines [98], this factor has been recognized as one of the most important adipokine. Adipocytes secrete TNF-*α*, and the expression of this factor is increased in the hypertrophied adipocytes of obese subjects. TNF-*α* is the molecule linking inflammation with obesity [99]. We will further discuss this adipokine in the diet-induced hypertension section.

### 4.5. Prostaglandins (Adipocyte Derived)

Prostaglandins, together with angiotensin II and endothelin-1, are the most vasoactive substances generated by adipocytes. Adipocytes produce prostaglandins in response to sympathetic stimulation. Lipolytic hormones, like adrenaline, are linked to the hypertensive status and obesity-associated hypertension. These hormones target membrane adipocyte *β* receptors and in turn activate hormone sensitive lipase. This stimulus induces lipolysis, release of fatty acids, and prostaglandins, especially PGE_2_ and PGI_2_, which are also fatty acids in origin. Antilipolytic stimuli, insulin, for example, reduce the release of prostaglandins [100] such as prostacyclin (PGI_2_). On the basis that insulin decreases the production of this strong vasodilator, Parker and coworkers suggested that hypertension associated with insulin resistance and hyperinsulinemia (i.e., metabolic syndrome) would be due partly caused by the lack of proper PGI_2_ release [69]. It appears that PGI_2_ production by the adipocytes results from the cooperation of adipocytes and vascular endothelial cells. Parker and coworkers proved that adipocytes are a source of the original fatty acid component of prostaglandins, arachidonic acid, that is converted into prostaglandins by the closely located vascular endothelial cells. Adipocytes provide arachidonic acid but lack the required cyclooxygenase which is provided by adjacent endothelial cells [69]. However, adipocytes do express cyclooxygenase [101], and according to Richelsen et al., adipocytes can synthesize prostaglandins, but still provide endothelial cells with adipocyte-derived arachidonic acid to further generate prostaglandins [100]. 

### 4.6. Angiotensin II (Adipocyte Derived)

The first to propose PVAT as a source of angiotensin II were our previously quoted Soltis and Cassis who suggested that adipocyte-derived angiotensin II would favor vasoconstriction [71]. This effect could be due to the fact that the angiotensin II action prevents PI3K activation, resulting in a loss of stimulation of NO synthesis by this route [102], as discussed in the section related to endothelin-1. Plasma renin activity and thus the production of angiotensin II are high in obese individuals [5, 19]. Three possible explanations have been proposed to explain this phenomenon: (1) obesity may raise renin secretion by increasing loop of Henle sodium chloride reabsorption and reduce sodium chloride delivery to the macula densa [19]; (2) obesity may stimulate renin secretion by activation of the sympathetic nervous system [19]. Finally, (3) the existence of a high renin activity in the hypertrophied adipocytes causing an increased angiotensin II release [103–106]. Today, we know that adipocytes possess the whole enzymatic machinery involved in the renin-angiotensin system [103] and, in fact, they do synthesize angiotensin II [105, 107]. Importantly, angiotensinogen gene expression is higher in intra-abdominal fat than in other fat depots or nonadipose tissues [108]. Indeed, increased production of angiotensinogen by intra-abdominal fat appears to explain the high circulating levels of this peptide observed in dietary obesity [104]. Closely related with the physiology of angiotensin II is aldosterone. The levels of this corticoid are elevated in some obese hypertensives, especially patients with visceral obesity [109]. Furthermore, it has been recently discovered that adipocytes also produce aldosterone (actually in response to angiotensin II) [106]. In this regard, the adipocyte may be considered a miniature renin-angiotensin-aldosterone system.

It is noteworthy that adipose cells also secrete mineralocorticoid-releasing factors with important effects on aldosterone release from adrenocortical cells [110]. These are called *adipogensins* or aldosterone-releasing factors (ARF) [111] but are not well characterized as yet. There is a lot of data that suggests a close relationship between an excess in released aldosterone and insulin resistance. Aldosterone promotes insulin resistance through mineralocorticoid receptors activation (independently of gene transcription) in a large number of tissues [112]. On the other hand, hyperinsulinaemia induces increase in aldosterone levels [113, 114] thus creating another positive feedback cycle between hyperaldosteronism and hyperinsulinemia, with important pathophysiological effects in subjects with insulin resistance and a potential mechanism for the development of complications in obese hypertensive patients. 

### 4.7. Endothelin-1 (Adipocyte Derived)

As stated in previous lines, endothelin-1 is a vasoconstrictor protein normally produced by the endothelial cells but qualifies as adipokine as well [115]. Indeed, the levels of endothelin-1 increase in obesity and type II diabetes [116, 117]. In studies of experimental obesity, an increase in endothelin-1 gene and protein expression has been detected within the cardiovascular system [118]. Harmelen et al. found that obese adipose tissue releases 2.5 times more endothelin-1 than the adipose tissue of lean individuals. Furthermore, this ET-1 generates insulin resistance specifically in visceral, but not in subcutaneous, adipose tissue [119]. This links directly endothelin-1 with insulin resistance and obesity.

## 5. Animal Models of Metabolic Syndrome: Vascular Function

### 5.1. The Zucker Obese Rat

The Zucker rat is probably the most commonly used rat model for metabolic syndrome. In 1961, L. M. Zucker and T. F. Zucker discovered that an autosomal recessive mutation in the fatty gene (fa) resulted in obesity [120]. The homozygotes for the mutation (fa/fa) develop obesity because of a defective leptin receptor [121, 122]. Zucker rats develop insulin resistance in addition to obesity, but glycemia remains normal, and they do not develop diabetes [123]. In this aspect, the Zucker rat shares similarities with some of the obese subjects, those who are obese and insulin resistant but are not diabetic. However, the Zucker fatty rat does not mimic the cardiovascular, renal, and neurohumoral changes found in obese humans. For example, this rat has decreased plasma renin activity [124], whereas obese humans often reveal increased renin activity [5]. Also, increased sympathetic activity appears to play a significant role in causing hypertension in obese humans [125], but not in Zucker fatty rats [124]. In addition, conflicting results about whether obese Zucker rats are hypertensive or not compared with their lean controls have been repetitively reported [126]. In a carefully performed study by Hall's group, it was shown that obese Zucker rats suffer no more than 14 mmHg higher than the lean counterparts and that this depends in part on angiotensin II [124].

Regarding vascular responses, much work has been performed in aorta [127–131] and in resistance arteries [132–137] of Zucker rats. Endothelial function assessed in aortic preparations appears to be preserved, or even increased, in young Zucker obese rats compared to the lean rats [128–131]. Andrews et al. use the term *endothelial hyperreactivity *[130] to emphasize the superior endothelial function of Zucker obese rats [131]. For Auguet et al. the increased influence of endothelium in Zucker rats would be related to the absence of atherosclerosis (despite hypercholesterolemia) of these rats. As for resistance arteries, the majority of studies indicate impaired endothelial dysfunction [134–136] and impaired NO-dependent vasodilation [133, 137] in Zucker obese rat arterioles compared to the lean counterpart. By contrast, one study finds equal endothelial function [132].

### 5.2. The Spontaneously Hypertensive Obese (SHROB) Rat

The obese spontaneously hypertensive rat (SHROB), also known as Koletsky rat, is a rat strain of spontaneous hypertension breeding origin that suffers a nonsense mutation of the leptin receptor gene [138]. This animal was obtained by mating a female SHR of the Wistar-Kyoto strain with a normotensive Sprague-Dawley male. The resulting hybrid offspring was inbred and the obese rat appeared after several generations. The obesity mutation is a recessive trait, designated *fa* ^*k*^, which is a nonsense mutation of the leptin receptor gene resulting in a premature stop codon in the leptin receptor extracellular domain. The SHROB ratcarries two *fa* ^*k*^ alleles; it is leptin resistant and has circulating leptin levels 30 times higher that the lean counterpart. This mutation makes SHROB rats unable to respond to leptin [139, 140]. This strain arose spontaneously in 1969 in Koletsky's laboratory in Case Western Reserve University School of Medicine (Ohio) [141]. The rat displays obesity, hypertension (although milder than that of their SHR ancestor), hyperinsulinaemia, hyperlipidaemia, and nephropathy, all superimposed on a hypertensive background. Thus, these rats exhibit all the symptoms of metabolic syndrome and are generally regarded as an adequate animal model of this disease [126]. 

Cardiovascular and renal function has been hardly explored in the SHROB rat. Still, it is known that SHROB rats develop a pronounced diabetic retinopathy. This makes them of special interest for the study of the microvascular complications associated with metabolic syndrome. Huang and coworkers noted that already at 3 months of age they displayed very mild microvascular alterations and did not develop diabetic retinopathy until 10 months of age. Interestingly, control lean SHROB rats also develop diabetic retinopathy [142]. 

The effect of diet on blood pressure changes has also been studied in these animals. Ernsberger and coworkers observed that drastic fluctuations in the supply of nutrients are not beneficial for blood pressure in these animals. They show that restrictive diet followed by feedback cycles produces blood pressure elevations caused by sympathetic activation and cardiac hypertrophy [143]. 

Regarding renal and cardiovascular function, it is known that specific binding sites for angiotensin II are decreased in SHROB rats with early glomerular sclerosis, suggesting that angiotensin receptors may be regulated by pathogenic processes in kidneys of these animals [144].

**Figure 2:**
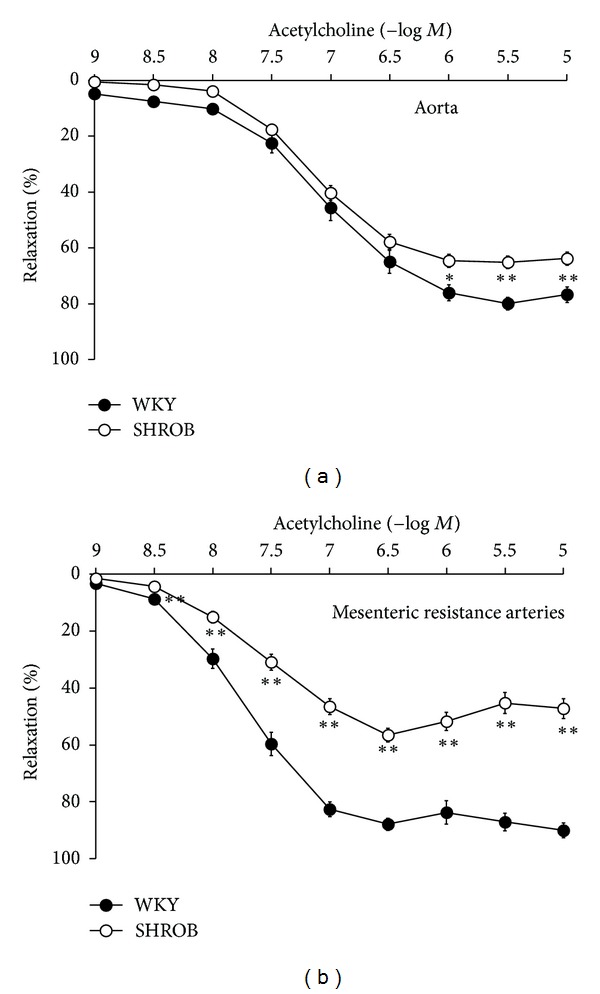
Endothelial function tested by means of acetylcholine responses in aorta (a) and resistance arteries (b) of normotensive (WKY) and metabolic syndrome rats (SHROB). Modified from Mendizábal et al. [145].

Recently, our group has characterized the macrovascular and microvascular function of this rat strain and the effects of a kind of antidiabetic drugs, glitazones, used in the handling of metabolic syndrome [145]. The SHROB rat clearly suffers macrovascular and most especially microvascular dysfunction (Figures 2(a) and 2(b)). Mesenteric resistance arteries of SHROB rats display a severely impaired endothelium-dependent relaxation due to a failure in the NO system and an abnormally high release of vasoconstrictive prostanoids. These rats also exhibit a dramatic loss in endothelium-independent relaxation, specifically to exogenous NO, suggesting a malfunction of guanylate cyclase. We also showed that drugs used for metabolic syndrome therapy, glitazones, have salutary effects on the endothelial dysfunction of these rats. 

### 5.3. The JCR-LA-cp Corpulent Rat

The JCR-LA-cp corpulent rat is another rat model used to study metabolic syndrome. This rat is homozygous for the autosomal recessive *cp gene (cp/cp)* and is obese, hyperphagic, insulin resistant, hyperinsulinemic, and hypertriglyceridemic [146]. In addition, male JCR-LA-cp rats develop atherosclerosis and myocardial ischemia. Vascular responses and endothelial function were studied by O'Brien and coworkers [146] rendering similar results as for micro- *versus *macrovascular endothelial dysfunction as those of SHROB rats, although the latter displayed a more intense impairment of acetylcholine responses. 

### 5.4. Diet-Induced Obesity

*Stricto sensu*, this model of obesity cannot be always categorized as an animal model of metabolic syndrome because dieting an animal with high fat chow rarely causes the complete cardiovascular and metabolic disease. In some cases, obesity-induced hypertension is achieved [147, 148], but this is not commonplace and most research papers do not report blood pressure values. Other metabolic syndrome symptoms are irregularly reported. For example, hyperinsulinemia or hyperglycemia is found in some studies [149, 150] but not in others [151, 152]. Dyslipemia takes place in some [149, 151] but not all the studies [152]. Hyperleptinemia seems to be common to all [150–152]. 

However, keeping in mind the enormous epidemiological dimension of overweight, obesity, and obesity-associated cardiovascular problems (i.e., cardiometabolic syndrome), much research and effort have been performed in these kind of rat or mouse models regardless of whether the animal develops or not a complete metabolic syndrome. Another factor in favor of diet-induced obesity animal models is that they are more human-like models, where the obesity is based on an excess intake of calories, whilst genetic models deficient in the leptin receptor or leptin synthesis are not representative of the human pathophysiology of obesity. Obesity in rodents can also be induced with the so-called *cafeteria diet*. In this model, animals have a choice of various energy-dense foods. The advantage to this approach is that the diet is palatable and the propensity to overeat is larger than that for the high-fat chow diet. Needless to say is that this is the most similar to the human dietary situation [153]. 

Regarding vascular function, the vast majority of studies have reported alterations. Endothelial function, assessed by acetylcholine responses, has been found altered in most cases. For example, in a cafeteria diet model reported by Naderali et al., a negative association between plasma lipid levels and reduction in acetylcholine-induced vasorelaxation was found [151]. Furthermore, a study in obese people showed that weight loss improves endothelial function together with various metabolic syndrome symptoms [154]. Hypercontractility, albeit less studied, has been reported in rats made hypertensive through the diet [147, 148]. In recent times, a large amount of studies have been focused on the effects that the local adiposity surrounding blood vessels (the so called PVAT) has on smooth muscle cell contractility and endothelial function [77]. Adipose tissue specifically located close to blood vessels exhibits a proinflammatory phenotype compared to other depots such as the subcutaneous one [155]. This phenotype is aggravated after a high fat feeding suggesting that PVAT is very sensitive to the effects of excess dietary fat [155]. In obese rats, including diet-induced obesity rats, it has been repetitively shown that PVAT causes endothelial dysfunction via proinflammatory cytokines such as TNF*α* [156] or monocyte chemotactic protein-1 [157] as well as through oxidative stress [148, 157]. Actually, Dobrian et al. report on a rat model of obesity-induced hypertension that this increase in vascular oxidative stress is associated with an increase in vascular NO production and NO synthase activity [148]. Furthermore, Jebelovszki et al. demonstrated that diet-induced obesity increases vascular smooth muscle sensitivity to NO through an activation of guanylate cyclase [149]. To have a whole picture of the biology of NO in obesity it is important to consider also that adipocytes can express NO synthase and that this expression is upregulated in obesity [158]. The adipocytic upregulation of NO synthase contrasts with the endothelial downregulation of this enzyme described by Ma et al. in diet-induced obesity rats in which this downregulation finely correlates with the vascular dysfunction they find in their own experiments [159] and, in general, in those of others [152–157]. This apparent contradiction can be explained as follows. In nonobese individuals, PVAT would have a vascular protective and beneficial role [152]. During the onset of obesity, several adaptive mechanisms within the vessel wall [149] and within PVAT itself [152] are activated. Regarding the latter, Gil-Ortega et al. have published interesting data showing the existence of an adaptive NO overproduction by PVAT during early diet-induced obesity and propose that, at some time point during obesity development, PVAT switches from a vascular protective influence to a deleterious one [152].

## 6. Future Directions

The pathophysiology of metabolic syndrome has become very complex. We have reviewed some of the pathophysiological aspects that affect vascular function: insulin, sympathetic system, endothelium, perivascular fat, and adipokines. The animal models in use have important limitations that need to be compensated with clinical studies. Translational research, in which animal studies are designed and carried out together with clinical investigation, is of special value. It is also highly important to merit basic science studies designed to unravel specific pathways, messengers, and intermediates of metabolic syndrome. While the era of endothelium and endothelium-derived substances has passed its summit, the age of perivascular adipocytes and adipokines is coming with a strong impulse.

## Figures and Tables

**Table 1:** Endocrine and vascular paracrine functions of some adipokines.

Adipokine	General effects	Vascular effects	References
Leptin	Satiating factor Physiological regulation of feeding behaviour through hypothalamic receptors Levels correlate with amount of body fat	Endothelial dysfunction Endothelium-dependent and independent relaxation	[67, 85, 160–165]

Resistin	Relates obesity to diabetes by inducing insulin resistance	Impairs endothelial function due to an increase in ET-1 production and a decrease in NO production	[166, 167]

Adiponectin	Levels inversely correlate with obesity	NO-dependent vasorelaxation mediated by *K*_*v*_ channels	[91, 93, 94, 168]

Visfatin	Expression correlates with obesity degree Similar effects to insulin in cell culture	NO-dependent vasorelaxation	[169–171]

TNF*α*	Links inflammation with obesity Increase in TNF*α* expression induces ROS production Reduces adiponectin production	Endothelium-dependent and -independent vasodilatation Triggers ET-1 and Ang II-induced vasoconstriction Impairs endothelium-dependent vasodilatation due to increased ROS production or decreased NO production Less vasodilatory effect of PAT due to ROS production	[94, 99, 172–178]

Interleukin-6	Contributes to systemic inflammation and insulin resistance	Endothelium-independent vasodilatation Endothelial dysfunction due to an increase in ROS production and decreased NO production	[94, 179–182]

Prostanoids	See vascular effects Hemostasis Numerous biological functions	Vasoconstriction or vasodilatation depending on which prostanoid	[183, 184]

Angiotensin II	See vascular effects Na^+^ and water homeostasis Renal function	Vasoconstriction	[185, 186]

Endothelin-1	See vascular effects	Vasoconstriction	[187]

Reactive oxygen species	Numerous biological effects Ageing	Vasoconstriction through Ca^2+^ sensitization Decrease in NO bioavailability	[73, 188, 189]

Adventitial derived relaxing factor	See vascular effects	Vasorelaxation through opening different K^+^ channels	[75, 76, 78–80]
